# External Volume Expansion Modulates Vascular Growth and Functional Maturation in a Swine Model

**DOI:** 10.1038/srep25865

**Published:** 2016-05-13

**Authors:** Huang-Kai Kao, Hsiang-Hao Hsu, Wen-Yu Chuang, Sheng-Chih Chen, Bin Chen, Shinn-Chih Wu, Lifei Guo

**Affiliations:** 1 Department of Plastic and Reconstructive Surgery, Chang Gung Memorial Hospital & Chang Gung University College of Medicine, Tao-Yuan, Taiwan; 2 Kidney Research Center, Department of Nephrology, Division of Critical Care Nephrology, Chang Gung Memorial Hospital & Chang Gung University College of Medicine, Tao-Yuan, Taiwan; 3 Department of Pathology, Chang Gung Memorial Hospital & Chang Gung University College of Medicine, Tao-Yuan, Taiwan; 4 Department of Animal Science, National Taiwan University, Taipei, Taiwan; 5 Department of Plastic Surgery, Lahey Hospital & Medical Center, Burlington, MA, USA

## Abstract

Despite increasing application of the pre-grafting expansion during autologous fat transplantation in breast reconstruction, little is known about its mechanism of action. To address that, ventral skins of miniature pigs were treated over a 10-day or 21-day period, with continuous suction at −50 mm Hg via a 7-cm diameter rubber-lined suction-cup device. Soft tissue thickness increased immediately after this external volume expansion (EVE) treatment, such increase completely disappeared by the next day. In the dermis and subcutaneous fat, the EVE treated groups showed significant increases in blood vessel density evident by CD31 staining as well as in vascular networks layered with smooth muscle cells when compared with the control group. This finding was corroborated by the increased percentage of endothelial cells present in the treatment groups. There was no significant difference in the percentages of proliferating basal keratinocytes or adipocytes, nor in epidermal thickness. Moreover, the EVE had no effect on proliferation or differentiation potential of adipose stem cells. Taken together, the major effects of EVE appeared to be vascular remodeling and maturation of functional blood vessels. This understanding may help clinicians optimize the vascularity of the recipient bed to further improve fat graft survival.

The use of external negative pressure has long been theorized as a means to create or expand tissues. In 2000, Khouri introduced an external negative tissue expansion system for nonsurgical breast enlargement^1^. Despite initial promising clinical results, limitations on the amount of volume expansion, as well as difficulties with patient compliance, have led to some dampened enthusiasm. The benefits of vacuum assisted closure (VAC) on wound healing and tissue regeneration beg for the hypothesis that tissues treated with external negative pressure would be able to support larger volumes of fat grafting than previously thought possible^2^. The subsequent success of “mega-volume” fat grafting in clinical trials introduced a new method of breast reconstruction and augmentation^3^.

As is common with new techniques, there has been significant debate, regarding both the methods of quantifying the results and the underlying physiologic processes^4^,^5^. Studies based on a mouse model demonstrated an increase in both vascular proliferation and subcutaneous fat (without grafting)^6^,^7^. The clinical applicability of the results from a murine model, however, has been debated largely out of underwhelming clinical experience of external negative pressure without fat grafting. The discrepancy between findings in mice and human has been attributed to their innate anatomical, physiologic, and mechanical differences^8^.

New vascular network formation is critical for fat graft survival and tissue regeneration; however, the viability of fat grafting is dependent on nutrient diffusion and neovascularization, especially in the early phase after transplantation. The effects of external mechanical stimulation on vascular network formation and remodeling suggest that the neovascularization process depends on its surrounding environment and the magnitude of the stimulus^9^. The applied negative pressure using external volume expansion (EVE) may provide such a potential means of intervention to enhance vascularization of the recipient bed.

Previous studies showed effects of mechanical force on cell phenotype, growth rate, and signal transduction in a variety of cell types. These data also demonstrated that mechanical loading can induce osteogenic differentiation and smooth muscle differentiation in mesenchymal stem cells^10^,^11^,^12^. However, the assessment of affected tissue’s cellular compositions, adipogenic differentiation, and cellular markers in adipose stem cells (ASCs) isolated from EVE treatment has yet to be made.

In our current study, the effects of *in vivo* mechanical loading of EVE on neovascular growth and subsequent functional maturation of blood vessels in subcutaneous fat in a swine model were examined. We hypothesized that *in vivo* mechanical loading would modulate neovascular growth and vascular remodeling as a function of the timing of load application. Therefore, the EVE effects of short-time (10 days) and long-time (21 days) loading were herein quantitatively evaluated on vascular growth and remodeling in the subcutaneous fat. Moreover, we performed quantitative assessments to compare the effects of different treatment interval of EVE on swine ASCs in terms of cell phenotype, proliferation capacity, differentiation potential, and stemness. A swine model was chosen because of its skin architectural and physiologic similarities to human’s, thus likely more relevant to actual clinical experience than rodent models, from which most of the existing knowledge has been derived.

## Results

### EVE had no significant lasting effect on skin and subcutaneous thickness

An ultrasonography was performed to study the soft tissue thickness (of skin and subcutaneous fat) before EVE treatment, immediately after the last EVE treatment or before sampling at day 11 and day 22. There was no statistical difference between thickness measured before EVE treatment and thickness measured before sampling. However, soft tissue thickness measured immediately after treatments significantly increased as compared to the pre-treated thickness (15.7 ± 0.99 *vs*. 13.2 ± 1.04 at day 10, p < 0.01; 15.9 ± 1.02 *vs*. 13.24 ± 0.99 at day 21, p < 0.01) (Table 1). EVE treated areas demonstrated gross local swelling immediately after suction procedures at day 10 or day 21, which subsided by the next day before sampling at day 11 or 22. These results indicated that the EVE induced soft tissue enlargement is a transit effect even after it was applied for 21 days.

### EVE enhances vascular remodeling and maturation but not adipogenesis in subcutaneous tissue

**Figure 1:**
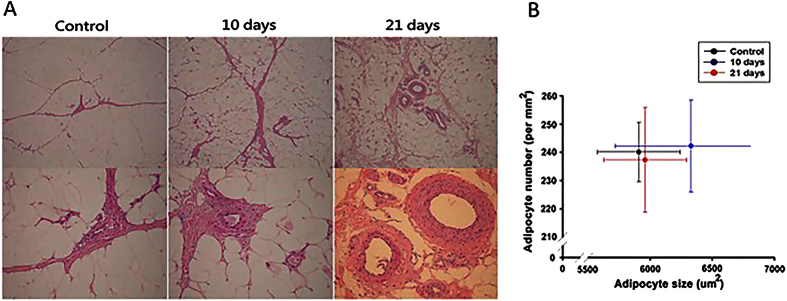
Histomorphometrical changes of subcutaneous fat. (**A**) In EVE treated groups, the vascular networks in subcutaneous fat were stabilized by progressive lumenization and specialization of vessel wall for structural support (magnification; upper panel: 40Χ, lower panel: 200Χ). (**B**) Measurement of adipocyte size and numbers using Adiposoft software. There was no difference between the control groups and EVE treated groups.

The histomorphological analysis of H & E stain revealed the process of vascular remodeling was enhanced by EVE treatment. In EVE treated groups, the vascular networks were stabilized by progressive lumenization and wall thickening (Fig. 1A). There was no statistical difference in adipocyte size and numbers between the control and EVE treated groups (Fig. 1B).

### EVE promotes angiogenic response in dermis and subcutaneous fat

**Figure 2:**
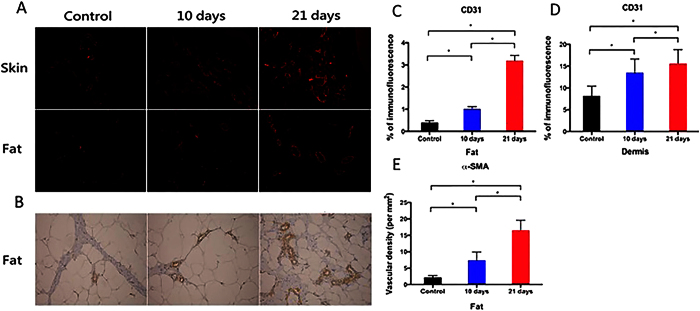
(**A**) Immunofluorescence stain with PECAM-1 (CD31) for neovascularization in dermis and subcutaneous fat (magnification: 200Χ). (**B**) Immunohistochemistry stain with α-SMA for vascular networks in subcutaneous fat (magnification: 200Χ). (**C,D**) In dermis and subcutaneous fat, the EVE-treated groups showed significant increases in calculated blood vessel density when compared with the control groups (*n* = 6 per group; *p < 0.05). (**E**) The EVE-treated groups demonstrated a significant increase in vascular networks layered with α-SMA (*n* = 6 per group; *p < 0.05).

CD31 (PECAM-1) was selected to stain for endothelial cells (Fig. 2A). In dermis and subcutaneous fat, the EVE treated groups showed significant increases in blood vessel density when compared with the control group. Furthermore, the EVE treated groups at 21-days had significantly higher CD 31 positive areas than at 10-days in both dermis and subcutaneous fat (Fig. 2C,D). The EVE treated groups also demonstrated a significant increase in vascular networks layered with smooth muscle cells. Specifically, the 21-day EVE treated groups had approximately 16.8 blood vessels per square millimeter, whereas only 7.1 and 2.5 blood vessels were found per square millimeter in the 10-day EVE treated and control groups, respectively (Fig. 2B,E).

### EVE had no significant effect on cell proliferation in skin and subcutaneous fat

**Figure 3:**
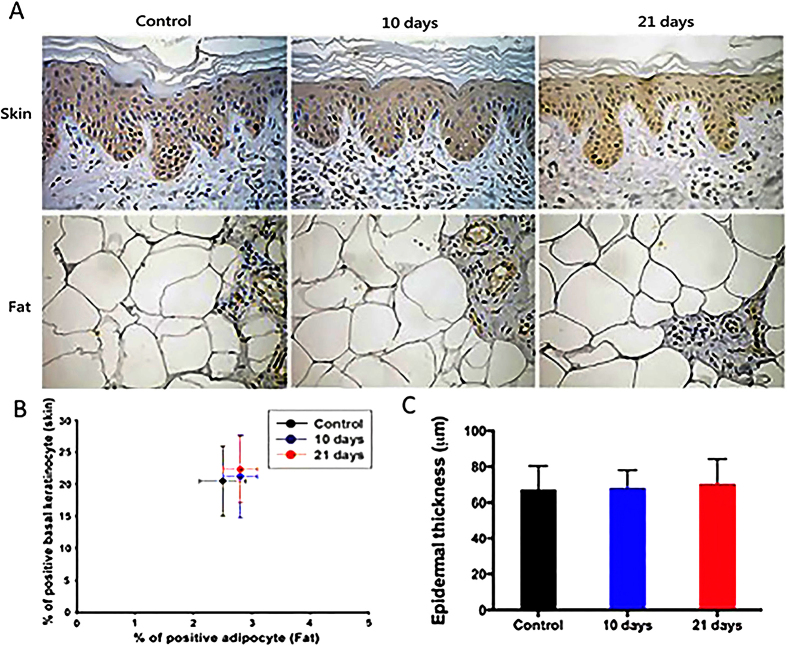
(**A**) Immunohistochemistry stain with Ki67 for cell proliferation in basal keratinocytes and adipocytes (magnification: 200Χ). (**B**) There was no difference in the percentage of basal keratinocyte and adipocytes labeled with Ki67 (*n* = 6 per group; *p < 0.05). (**C**) No difference in epidermal thickness across all groups.

The proliferation rates of basal keratinocytes in skin and of adipocytes were assessed by nuclear staining for Ki67, a universally accepted proliferation marker (Fig. 3A). There was no statistical difference in the percentage of Ki67-labeled basal keratinocytes and adipocytes (Fig. 3B). Nor was there a statistical difference noted in epidermal thickness across the groups (Fig. 3C).

### EVE had no significant effect on proliferation and differentiation potentials of ASCs

**Figure 4:**
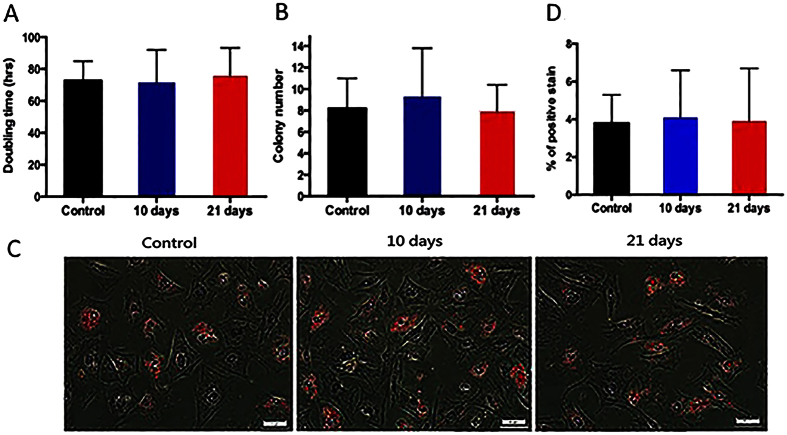
Proliferation and differentiation potentials of adipose stem cells (ASCs) from control and EVE treated groups. (**A**) Doubling time of P0 ASCs. No difference was noted across all groups. (**B**) Number of colonies formed after 7 days of culture at initial plating of 5000 ASCs for each group. (**C,D**) Adipogenic differentiation (Oil-Red O stain) in ASCs of each group responding to differentiating medium after 21 days. Differentiation levels varied between groups but not significantly as evident by colorimetrical evaluation of Oil-Red O uptake from counting Oil-Red O positive cells.

The proliferation rate of passage zero (P0) ASCs was evaluated by measuring their average doubling time. There was no significant difference in the doubling time across the groups (Fig. 4A). Proliferation of ASCs was also measured by a colony forming assay in which 5000 P0 ASCs were plated and their formed colonies were counted as well as the number of cells in each colony after 7 days of culture. The formed colonies (>20 cells per colony) for each group were measured. As shown in Fig. 4B, no statistical difference was observed between the control and EVE treated groups. To test the adipogenetic differentiation potential of ASCs, cells were subjected to adipogenic induction for 21 days followed by Oil-Red O staining. Again, there was no statistical difference in the percentage of positive Oil-Red O cells (Fig. 4C,D) across all the groups.

### EVE induced increase in the ratio of endothelial cells of stromal vascular cells

**Figure 5:**
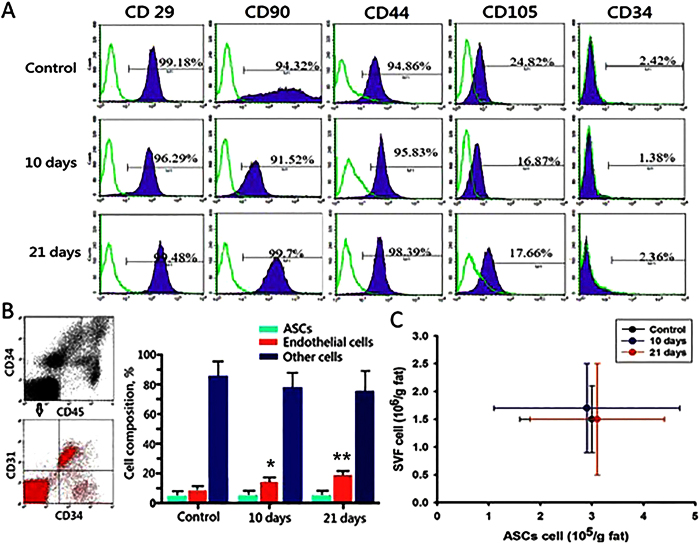
(**A**) Immunophenotype of adipose stem cells (ASCs) of each group revealed very similar expression patterns of surface markers. Flow cytometry analysis showed the expressions of CD29, CD44, CD90, and CD105 were at high levels and the expression of CD34 was at a lower level. (**B**) Representative plotted data of SVF cells using multicolor flow cytometry analysis. CD45^+^ cells were regarded as blood-derived cells, whereas CD45^–^ cells were regarded as ASCs and processed to the next analysis. CD45^–^CD31^–^CD34^+^ cells, CD45^–^CD31^+^ CD34^+^ cells, and CD45^–^CD31^–^CD34^–^ cells were regarded as ASCs, endothelial cells, and other cells (fibroblasts, mural cells, and others), respectively. No difference in the ratio of ASCs across the groups. The percentage of endothelial cells of EVE treated groups significantly increased when compared to the control group (8.1 ± 1.9% in the control groups, 15.2 ± 3.0% in the 10-day treated groups, and 20.3 ± 3.8% in the 21-day treated groups) (*n* = 6 per group; *p < 0.05 *vs*. control, and **p < 0.01 *vs.* control & *p* < 0.05 *vs*. 10-day treated groups). (**C**) No difference was noted in the total number of ASCs and stromal vascular fraction (SVF) cells.

Flow cytometry analysis showed that ASCs exhibited CD29, CD90, CD44, and CD105 at a relatively high levels and CD34 at very low levels. A representative phenotypic analysis of ASCs is shown in Fig. 5A, demonstrating the homogeneity of the phenotype and very similar expression patterns of surface markers. There were no differences in the total number of stromal vascular cells and ASCs across the groups (Fig. 5C). Multicolor flow cytometric analysis of stromal vascular cells revealed no difference in the ratio of ASCs at 5.0 ± 1.9% in the control groups, 5.6 ± 2.5% in the 10-day treated groups, and 4.8 ± 2.3% in the 21-day treated groups. In contrast, the percentage of endothelial cells of EVE treated groups significantly increased as treatment lengthened when compared to the control group (8.1 ± 1.9% in the control groups, 15.2 ± 3.0% in the 10-day treated groups, and 20.3 ± 3.8% in the 21-day treated groups) (Fig. 5B).

## Discussion

Nonvascularized fat grafting is an established procedure for soft tissue augmentation; however, clinical outcome following fat grafting remains variable and technique-dependent^13^. Despite increased application of a pre-grafting expansion device called Brava (Brava LLC, Miami, FL) during autologous fat transplantation in breast reconstruction and augmentation, little is known about its mechanism of action and how the graft is revascularized. In the current study, a novel experimental swine model was devised to test the preconditioning effects of EVE on the subcutaneous potential grafting recipient site. The miniature pig skin model was chosen because it is much more similar to human skin than other related published animal models. The principal experiment results showed that: (1) neovascular network formation, growth, and maturation of functionalized blood vessels in adipose tissue were the major effects of external volume expansion. (2) the mechanically stretched condition *in vivo* modulated and stimulated vascular growth and remodeling. (3) the EVE induced soft tissue enlargement was transient even after 21 days of daily treatments. (4) EVE had no significant effect on proliferation and differentiation potential of ASCs.

Remodeling of adipose tissue after fat grafting is a dynamic process. Revascularization of fat grafts is initiated by plasmatic diffusion from surrounding tissues until vascular network ingrowth from the recipient site^14^.A well-vascularized recipient bed with early and adequate revascularization of fat graft is pivotal to its survival^15^,^16^. The impact of vacuum assisted closure (VAC) therapy on angiogenesis associated with wound healing has been investigated largely through the establishment of gradients in hypoxia and vascular endothelial growth factor and the consequent activation of endothelial cell proliferation^17^,^18^,^19^. Thus,these observations may be extrapolated to lead to the hypothesis that the same mechanism as VAC to enhance angiogenesis could be applied in EVE treated intact soft tissue. Recently, Heit *et al*. developed a miniaturized EVE device in a murine model, using continuous suction with negative pressure of 25 mmHg. In that study, the treated group showed a 1.9-fold increase of vascular density in the subcutaneous fat layer when compared to the untreated group^6^. Furthermore, they concluded that macroscopic tissue stretch could induce local ischemia, which could trigger inflammation and, either independent of or through inflammation, could activate pathways leading to angiogenesis and vessel remodeling.

To better understand the effect of EVE on angiogenesis in a transplanted bed, a swine model was devised to take advantage of anatomical and physiological similarities in skin and subcutaneous tissue between pig and human. In the present study, the EVE treated group showed significantly greater vascularity in the subcutaneous adipose tissue than the control group as demonstrated by CD31 (PECAM-1) staining. The EVE treated group also showed an increase in relatively mature and sprouting vascular networks evident with more smooth muscle cells and significantly larger vessel diameters. This is consistent with the multicolor flow cytometric analysis of stromal vascular fraction (SVF) cells, in which the EVE treated groups contained a significantly higher percentage of endothelial cells. It is therefore possible to speculate that the application of EVE resulted in microdeformation of adipose tissue, which enhanced angiogenesis, neovascularization, and vascular remodeling. Our results are consistent with findings from the murine model and with observations of the effects negative pressure therapy had on wound healing.

Furthermore, within the EVE treated group comparing day 10 to day 21, there was clear evidence of a steady development in vascularization, lumenization and lumen maturation with the thickened muscle layer of blood vessels as the EVE treatments wore on. The results suggested that in face of externally applied negative pressure, angiogenesis, vascular growth, and maturation of functional blood vessels in both dermis and subcutaneous adipose tissue responded in a time- and dose-dependent manner.

Adipose tissue expandability in response to external mechanical stimulation has not been well addressed in the literature. In a murine model, it was shown that EVE could increase subcutaneous thickness as well as cell proliferation in the subcutaneous fat layer^6^. From those data, it was proposed that the adipogenetic role of localized edema may explain the mechanism of action of EVE on subcutaneous tissue^7^,^8^. Edema has been described as a possible mechanism involved in adipogenesis. Increased fluid in the interstitial space, which can regulate the expression of adipogenetic factors, leads to changes in tissue composition with thickening of the skin and subcutaneous tissue^20^,^21^. A link between inflammation and adipogenesis is probably responsible for soft tissue overgrowth observed in lymphedema^22^,^23^. However, lymphedema is a chronic condition and the pathophysiology of its adipocytic expansion may bear little resemblance to acute effects of negative pressure treatment. It has been argued that tissue edema is only a transitory phase during a negative-pressure treatment and may not result in fat augmentation^24^.

In the present study ultrasound measurement of tissue thickness was performed. Our results revealed an average of 19% increase in tissue thickness immediately after eight hours of external negative pressure; however, in all cases this effect had dissipated gradually after the discontinuation of treatment even by the time of the next treatment 16 hours later (Table 1). Skin tissue measured by ultrasound at that point showed no increase in tissue thickness. These results indicated that EVE-induced soft tissue enlargement is a rapid, self-reversible, and transient process at least within the 21-day duration of our study. This notion is further corroborated by our proliferation assay using Ki67 as a marker. In our study, the Ki67 positive keratinocytes and adipocytes were identified and quantified in the epidermis and subcutaneous fat layer respectively. The soft tissue treated with EVE showed a slightly increased percentage of Ki67 positive cells in both the epidermal and adipose layer when compared with the non-treated group, however, this increase did not appear statistically significant. In Khouri’s early experience using BRAVA without fat grafting, the breast size increased 98 ± 67% over the initial size after a 10-week treatment course and achieved 55% increase at the 30-week follow up. In addition, in a most recently published study by Lujan-Hernandez *et al*., adipogenesis was demonstrated with perilipin-A staining in a mouse model after two hours of daily external volume expansion^25^. Interestingly, in human, it was postulated that the early volume increase was reversible with no tissue growth^24^. It was pointed out in Khouri’s original study in 2000^1^ that it “is only after continued use and sustained stretch that true tissue growth is stimulated”. It is worth noting that, in reality, most clinicians use BRAVA-assisted fat grafting for breast augmentation instead of using BRAVA only. In our current swine model, no adipogenesis was found by Ki-67 staining and the discrepancy may be attributed to a relatively shorter treatment period. In a mouse model, where the skin is much looser and more pliable than skin of either human or swine, shorter treatment course may therefore have a more profound effect on potential adipogenesis. Mature adipocytes have been known not to proliferate^26^. Adipogenesis is a process by which undifferentiated precursor cells differentiate into adipocytes and requires close interplay with blood vessels and the extracellular microenvironment. With no recruitment of fat, our result revealed EVE had no significant effect on proliferation and differentiation potentials of ASCs within our treatment period. Taken together, this seems to suggest, at least in a swine model, that cell proliferation and neo-adipogenesis do not play as dominant a role as neoangiogenesis and vascular remodeling under the external volume expansion condition. Length of external volume expansion is clearly a parameter that deserves additional attention with conceivable clinical impact on its pre- and post-grafting usage. Future study may be necessary to elucidate whether a much longer external expansion course, in human-like skin such as in a swine model, may lead to true onset of adipogenesis and how grafted fat may react to external volume expansion in addition to its behavior in a better vascularized bed.

Conventional tissue expansion may result in epidermal hyperplasia and decreased dermal and subcutaneous fat thickness. Unlike conventional tissue expansion, there was no difference in epidermal thickness between the control and EVE treated groups. Microscopic finding was also consistent, showing no significant increase in the percentage of proliferating basal keratinocytes. The increased epidermal thickness during conventional tissue expansion is primarily because of a slow biological tissue creep induced by a continuous and chronic stretching force, whereas the mechanical effect of EVE on intact skin was rapid but short lived, in which soft tissue changes would return to the pre-expansion status over time following discontinuation of the expansion process. Separately, however, it seems that vascular networks in dermis and subcutaneous fat were more sensitive to the externally applied mechanical force than the matrix cells such as keratinocytes, fibroblasts, and adipocytes.

ASCs isolated from each group exhibited a fibroblastic morphology and phenotype common to MSC that was independent of treatment interval. That is, regardless of treatment interval, ASCs expressed CD44, CD29, CD90 and CD105 (mesenchymal markers) while lacking expression of CD34 (hematopoietic markers). The cellular components of adipose tissue include a large collection of adipocytes and SVF cells, which contained hematopoietic cells, endothelial cells, and ASCs. We also compared the proliferation potential and adipogenic differentiation of isolated ASCs as well as cell number of ASCs and SVF and found that the EVE treated groups displayed similar results as compared to control groups, suggesting that the proliferation and differentiation potentials of ASCs were not affected by the externally applied mechanical loading in the current model.

Cutaneous and subcutaneous similarities in not only anatomical architecture but also in pathophysiology between human and swine skin make our study clinically relevant. The parallel between our model and clinically applied BRAVA system is that a continuous negative pressure was applied in a cyclical treatment interval of eight hours per day during a 3-week period. There are increasing needs for mega-volume fat transplantation for various reconstructive procedures including breast augmentation and cancer reconstruction. Elucidating the dominant mechanism by which tissue responds to external volume expansion is important, as such insight would help clinicians optimize approaches to prepare a well-vascularized recipient bed to further improve fat graft survival.

Findings reported here highlighted the predominant mechanism of action of EVE, which would modulate vascular remodeling and maturation of functional blood vessels. The preconditioning effect of EVE has been demonstrated in the current swine model, which may be easily translated into clinical practices to enhance cell and tissue engraftment. This understanding may help clinicians optimize the vascularity of the recipient bed preoperatively to further improve fat graft survival.

## Methods

### Animal model and study groups

A total of three Lee-Song female pigs weighting 43 to 44 Kg aged 6 to 6.5-month-old were obtained from the Department of Animal Science, National Taiwan University (Taipei, Taiwan). This study was carried out in strict accordance with the recommendations in the *Guide for the Care and Use of Laboratory Animals* by the National Institutes of Health. All animals were used under approved animal protocols by the Ethics Committee of National Taiwan University (reference ID: NTU-100-EL-82) and housed accordingly. They were all under a standard anesthetic/ analgesic protocol for experimental procedures.

The pigs were positioned in a standing position in a limited space. A dome-shaped rubber device with a diameter of 7 cm and an internal volume of 140 ml was designed and connected to a suction pump (VAC Instill, KCI, San Antonio, Texas). They were treated with continuous suction at −50 mm Hg during the same eight-hour (9:00–17:00) interval each day. This pressure setting was chosen because it was the highest pressure pigs can tolerate with no immediately adverse biological response such as pain or erythema when the external volume expansion device was applied.

**Figure 6:**
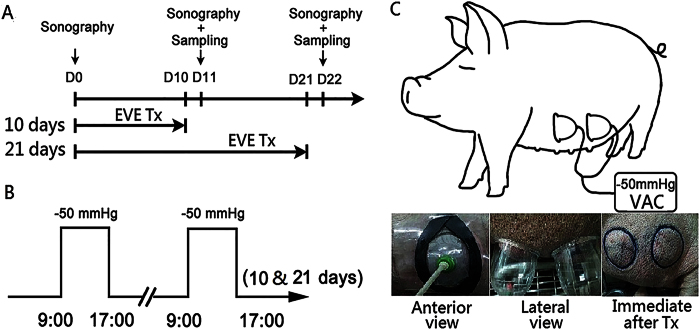
(**A**) Study groups and timechart of experimental design. (**B**) The treatment pattern. The EVE treated groups were subjected to a continuous suction at −50 mmHg during the same 8 hours (0900 to 1700) time intervals per day. (**C**) A dome-shaped rubber device with a diameter of 7 cm was designed and connected to a suction pump (VAC Instill, KCI, San Antonio, Texas) at a pressure of −50 mmHg. The device was applied to the ventral skin of the pig, 6 cm lateral to each single row of mammary glands. The suction procedure was conducted on two sites of each side at a time, with each site allocated to either a 10-days or a 21-days treatment time.

The device was placed to the ventral skin of the pig, 6 cm lateral to each single row of mammary glands. Suction was applied at two sites (front and hind, approximately 5 cm apart) on each side at a time. The mid-point between the front and hind sites was designated as the control site. All sites were, therefore, divided into three groups, with six sites per group across the three pigs: (1) control (without treatment) (2) EVE treatment for 10 days, and (3) EVE treatment for 21 days. For consistency and convenience, all front sites were allocated to a 10-day treatment period and all hind sites were allocated to a 21-day treatment period with control sites always between the front and hind sites across the three pigs. All tissue samplings took place a day after its respective treatment period ended (so on day 11 and 22 for the day 10 and 21 treatment groups) with the control group assayed on day 22 (Fig. 6).

### Ultrasonography measurement

Pigs were put in a lateral decubitus position and the measurement points for each group were marked. Soft tissue thickness, including skin and subcutaneous fat, was measured using an ultrasound unit with a 7.5 MHz linear-array real-time transducer (LOGIQ 700 MR, General Electronic Company, Milwaukee, WI, USA). The thickness was measured at 3 time points, including before treatment (day 0), immediately after the last treatment (day 10 or 21), and before sampling (day 11 or 22), all by a single operator.

### Histology and measurement of adipocyte size and number

Fixed tissue was dehydrated and embedded in paraffin blocks, from which 4-μm-thick sections were serially cut. Sections were stained with hematoxylin and eosin (H&E) as standard. All tissues described in this study were processed simultaneously to minimize artifacts. Bright-field whole-slide images were captured at 40Χ magnification (Plan-Neofluar objective with 0.50 NA) with an AxioImager.M1 microscope (Zeiss, Germany). The size and numbers of adipocytes were processed and analyzed automatically using Adiposoft software as described previously^27^.

### Immunohistochemistry for Ki67 and α-SMA

Paraffin-embedded sections were rehydrated and antigen retrieval for Ki67 was performed by microwaving in 10 mmol/L sodium citrate (pH 6.0) for 10 minutes. Frozen sections were fixed with acetone and stained for alpha smooth muscle actin (α-SMA) (Sigma-Aldrich, St Louis, MO). α-SMA primary antibodies were incubated at 4 °C overnight, whereas Ki67 (Lab Vision, Freemont, CA) primary antibody was incubated for 1 hour at room temperature. Signal was enhanced using the tyramide amplification system (PerkinElmer, Boston, MA).

### Immunofluorescence for CD31

Paraffin embedded formalin-fixed 3 μm-thick sections were deparaffinized, rehydrated, then antigen retrieval was performed by microwaving twice in 10 mmol/L sodium citrate (PH 6.0) for 10 minutes each. Bovine albumin solution (4%) in PBS was applied to block non-specific endogenous antigens. The unlabeled primary antibody detecting CD31 (Abnova, Cat # PAB11924, Taipei, Taiwan) was applied and incubated overnight at 4 °C. After three more 5-min PBS washes, fluorophore-labeled secondary antibodies were applied and reacted in the same manner as the primary antibodies. After another three 5-min PBS washes, the specimen was mounted and glass cover slips were attached for visualization under a fluorescent microscope (Axiovert 100, Zeiss).

### Quantification of blood vessel density & diameter

To quantify angiogenesis, three fields of each CD31 stained wound, were evaluated at 200Χ magnification. The neovascular area (CD31 positive cells) was measured using Image J and was expressed as a percentage of CD31 positive area to total image area. The number and diameter of vessels with a definite lumen were counted in each wound using immunohistochemistry stain for α-SMA. Each 1 mm^2^ area was counted and averaged for each slide in a blinded manner for statistical analysis with three randomly chosen fields.

### Quantification of cell proliferation& epidermal thickness

Proliferating adipocytes and epidermal cells (Ki67 positive) were counted separately at 200Χ magnification from respective sections. Proliferating adipocytes were evaluated in five high power fields. Within this area, the degree of proliferation was quantified over the entire section and expressed as a percentage of proliferating nuclei (Ki67 positive) to total nuclei. Only basal keratinocytes were counted as they are the only epidermal cells with proliferating potential. Three independent observers blinded to experimental treatment modes completed these analyses, and mean values of each measurement were taken for statistical analysis. The epidermal thickness at five evenly space points were measured and analyzed by Image J.

### ASCs isolation and adipogenic differentiation

The fat tissue was first minced into small pieces and were washed with phosphate-buffered saline (PBS) and digested on a shaker at 37 °C in PBS containing 0.075% collagenase (Wako Pure Chemical Industries, Osaka, Japan) for 30 minutes. Mature adipocytes and connective tissue were separated from pellets by centrifugation at 800 *g* for 10 minutes. The pellet was identified as the SVF cells. Portions of SVF were suspended and plated immediately in T225 flasks. The culture medium was M-199 medium containing 10% fetal bovine serum, 100 IU penicillin, 100 mg/ml streptomycin, 5 μg/ml heparin, and 2 ng/ml acidic fibroblast growth factor. This initial passage of the primary cell culture was referred to as passage 0 (P0). Adipogenic differentiation was induced as previously described using induction medium consisting of 10 μM dexamethasone, 0.25 μM 3-isobutyl-1-methyl- xanthine, 4 μM recombinant human insulin, 10 μM troglitazone, and 10% FCS^28^. Adipogenic differentiation was assayed by the formation of neutral lipid vacuoles stainable with Oil-Red O.

### Doubling time measurement and colony-forming assay

To measure the doubling time and colony-forming ability of ASCs, 5000 cells were initially plated in each well of 6-well plates and cultured for various periods using maintenance medium. A cluster of cells consisting of at least five cells was considered a colony. The number of colonies (>20 cells/ colony) formed by ASCs in each well was counted.

### Flow cytometry

For the analysis of surface marker expression, ASCs were washed three times with PBS, and then incubated with a blocking solution of 3% serum in PBS for 30 minutes. After centrifugation, 1 × 10^5^ cells were suspended in blocking solution, then stained with antibodies against human CD29, CD44, CD90, CD105, and CD34 (BD Biosciences). After incubation for 30 minutes, the cells were washed with PBS, and then analyzed by flow cytometry on a FACSCalibur (BD Biosciences, San Jose, CA) following standard procedures. In order to determine cell compositions of SVF cells, multicolor flow cytometry was performed with an LSR II (BD Biosciences, San Jose, CA). The following monoclonal antibodies conjugated to fluorochromes were used: anti–CD31-phycoerythrin, anti–CD34-phycoerythrin-Cy7, and anti–CD45-fluorescein isothiocyanate (BD Biosciences, San Jose, Calif.). Cell composition percentages were calculated according to data of surface marker expression profiles.

### Statistical analysis

Student’s t-test was used to test differences. Numerical data are expressed as mean ± SD. All tests were two-tailed, with a confidence interval of 95%. Normal distribution of data was tested using the Shapiro–Wilk test and equal variance was tested using the F-test. When the null hypothesis of normality and/or equal variance was rejected, the non-parametric Mann–Whitney U-test was used. Differences were deemed significant when *P* < 0.05. Statistical analyses were performed with SPSS version 13.0 (SPSS Inc., Chicago, IL).

## Additional Information

**How to cite this article**: Kao, H.-K. *et al*. External Volume Expansion Modulates Vascular Growth and Functional Maturation in a Swine Model. *Sci. Rep.* **6**, 25865; doi: 10.1038/srep25865 (2016).

## Figures and Tables

**Table 1:** Measurement of soft tissue thickness by ultrasonography.

	Average soft tissue thickness (mm)			*p* value	
	Pre-EVE	Immediate	Before	Immediate	Before sampling
		after EVE	sampling	*vs.* Pre-EVE	*vs.* Pre-EVE
10 days (n = 4)	13.13 ± 0.98	15.7 ± 0.99	13.2 ± 1.04	<0.01	0.38
21 days (n = 4)	13.24 ± 0.99	15.9 ± 1.02	13.3 ± 1.01	<0.01	0.41

EVE, external volume expansion.
